# Carbon Supported Gold Nanoparticles for the Catalytic Reduction of 4-Nitrophenol

**DOI:** 10.3389/fchem.2019.00548

**Published:** 2019-08-16

**Authors:** Hugo Rodríguez Molina, José Luis Santos Muñoz, María Isabel Domínguez Leal, Tomas Ramírez Reina, Svetlana Ivanova, Miguel Ángel Centeno Gallego, José Antonio Odriozola

**Affiliations:** ^1^Departamento de Química Inorgánica, Instituto de Ciencia de Materiales de Sevilla, Seville, Spain; ^2^Department of Chemical and Process Engineering, Faculty of Engineering and Physical Sciences, University of Surrey, Guildford, United Kingdom

**Keywords:** gold colloids, carbon, reduction, 4-nitrophenol, size effect

## Abstract

This work is a detailed study on how to optimize gold colloids preparation and their deposition to very different in nature carbon materials. The change of the continuous phase and its dielectric constant is used to assure the good dispersion of the hydrophilic/hydrophobic carbons and the successful transfer of the preformed small size colloids to their surface. The sintering behavior of the particles during the calcination step is also studied and the optimal conditions to reduce to a minimum the particle size increase during the protecting agent removal phase are found. The as prepared catalysts have been tested in a relevant reaction in the field of environmental catalysis such as the reduction of 4-nitrophenol leading to promising results. Overall, this work proposes an important methodology to follow when a carbonaceous material are selected as catalyst supports for green chemistry reactions.

## Introduction

Noble metal nanoparticles (NPs) remain one of the fast-growing fields in material science, due to their importance for optical, electrical, magnetic and catalytic technologies. All applications are based on nanoparticle's versatility due to their special size-morphology-properties relationship. Its massive use, however, is limited by geopolitical distribution and cost, disadvantages resolved by supporting preformed NPs on more sustainable materials such as polymers, organic molecules, inorganic structures and/or carbons. In this manner, NPs properties are preserved and the quantity of noble metal in the final material reduced (Goesmann and Feldmann, 2010; Tan et al., 2013; Chairam et al., 2017). Sometimes, NPs- support interaction occurs resulting in beneficial effect on material' overall properties. A good example is the integration of carbonaceous materials, i.e., graphene, carbon nanotubes, or activated carbons as supports for noble metals, where the support electrochemical, electrical, and catalytic properties are affected by the presence of metal (Tan et al., 2013). Activated carbon is a particularly important support for catalytic industry because of its high specific surface and stability in acidic/basic conditions and at moderate to high temperatures in low oxidizing atmospheres. Even more, its variable porous structure and low cost makes viable its use in many catalytic applications, especially in liquid phase reactions (Rodríguez-Reinoso, 1998).

An important part of the sustainability is the conversion of existing technologies, chemical industry in particular, in safer environmentally friendly processes. This could be achieved with the reduction of greenhouse gas emission and minimization of all toxic wastes. A worrying amount of hazardous wastewaters, generated daily, need expensive processing for minimization and/or transformation of all toxic residues to less dangerous products. Different pollutants such as dyes, heavy metals, or phenols and their derivatives are of great concern (Zhang et al., 2003; Chatterjee and Dasgupta, 2005; Menumerov et al., 2016). 4-nitrophenol (4-NP) is an example of harmful group of chemicals (phenols and derivatives) used in fungicides, medicines, dyes, and leather manufacture. It is known to be carcinogenic and mutagenic and causes skin diseases (Harrison et al., 2005; Zhao et al., 2010). Therefore, removal of 4-NP from waste water is key to ensure a healthy environment. However, its removal is a tedious process; physical, chemical, and biological treatments have to be used for this purpose.

The catalytic reduction of 4-NP in the presence of sodium borohydride (NaBH_4_) has emerged as a key process based on its simplicity, low cost and usefulness of the reaction product, 4-aminophenol (4-AP) (Du et al., 2004; Wunder et al., 2011; Hervés et al., 2012). 4-AP is non-toxic and widely used in pharmaceutical industry for analgesics and antipyretics, like paracetamol, phenacetin, etc. (Alif and Boule, 1991; Shi et al., 2005). This reduction is a catalytic process and supported noble nanoparticles are often reported as catalyst (Saha et al., 2009; Wunder et al., 2010). Among them, gold showed great selectivity and activity (Kuoda et al., 2009) and a special structure-size sensitivity that allows to study particles nitroarene hydrogenation activity and its proportionality to totally exposed particle surface (Kuoda et al., 2009; Lin and Doong, 2014).

To do this, an important size control is needed and referring to gold is not an easy task. One of the methods that allows a control over nanoparticles size and morphology is the colloidal route, where a gold colloid, freshly prepared, transfers to a support (Luo et al., 2013). To immobilize successfully the preformed colloids, we need to consider support nature and its hydrophilic/hydrophobic properties in particular for carbon based materials and its dispersability in the continuous phase.

For all described above, this work focuses on optimization of the gold colloids preparation and transfer to carbon based materials of different nature with the final aim to produce homogeneous and well dispersed gold nanoparticles based catalysts for 4-nitrophenol reduction in aqueous media. This work organizes around three approaches: (i) effect of solvent nature, (ii) effect of catalysts calcination parameters, and (iii) carbon nature influence on the catalytic properties.

## Experimental

### Synthesis of Au/C Catalysts

The experimental approach is based on the reduction of a gold precursor in the presence of stabilizing agent that allows particle size control. Once obtained the nanoparticles are transferred to the carbonaceous support (Megías-Sayago et al., 2018).

In a typical preparation, required amount of gold precursor (HAuCl_4_, Johnson Matthey, 49, 81% Au) was dissolved at room temperature in the continuous phase (water or water-ethanol mixture) with 5.10^−4^ M final gold concentration. Polyvinyl alcohol (PVA, Sigma Aldrich, 98%) 1% wt aqueous solution was used as stabilizing agent in PVA/Au = 0.85 weight ratio. After 20 min of continuous stirring, an adequate volume of 0.1 M NaBH_4_ (Sigma Aldrich, 98%) solution (NaBH_4_/Au = 10 molar ratio) was added to reduce rapidly the gold precursor and the resulting nanoparticles are kept under stirring for 20 more min. After, the colloid was immobilized on appropriate quantity of a commercially available activated carbon (CD) DARCO^®^ (Sigma Aldrich, 100 mesh particle size) to obtain 2% wt of gold in the final sample. The anchorage of the colloid on carbon surface was ensured by keeping both in contact until complete discoloration of the solution. Final suspension was filtered, washed with water or ethanol (depending on the solvent mixture used during the synthesis) dried at 100°C and finally calcined at 300°C.

Different water-ethanol mixtures were used as preparation media in order to study the effect of the solvent nature. Table 1 summarizes the solvent mixtures and the nomenclature of the resulting catalysts.

**Table 1:** Catalyst nomenclature and solvent mixture used in the synthesis.

**Catalyst**	**Water:ethanol % (v/v)**
Au/CD_100	100:0
Au/CD_70	70:30
Au/CD_50	50:50
Au/CD_30	30:70
Au/CD_8	8:92

For the optimization of the calcination step and PVA elimination, different calcination procedures were employed in order to avoid nanoparticles sintering (Table 2). For all methods, the temperature (300°C) and gas total flow (30 mL/min) remain constant.

**Table 2:** Tested calcination process over Au/CD_100 sample.

**Method**	**Catalyst**	**Time**	**Atmosphere**			**Heating rate (^**°**^C/min)**
			**Heating**	**Combustion**	**Cooling**	
1	Au/CD_100	2 h	S.A.	S.A.	S.A.	10
2	Au/CD_100	2 h	N_2_/S.A. (2% S.A.)	N_2_/S.A. (2% S.A.)	N_2_/S.A. (2% S.A.)	10
3	Au/CD_100	30 min	N_2_/S.A. (2% S.A.)	N_2_/S.A. (2% S.A.)	N_2_/S.A. (2% S.A.)	10
4	Au/CD_100	2 h	N_2_	S.A.	N_2_	10
5	Au/CD_100	2 h	N_2_	N_2_/S.A. (2% S.A.)	N_2_	10
6	Au/CD_100	30 min	N_2_/S.A. (2% S.A.)	N_2_/S.A. (2% S.A.)	N_2_/S.A. (2% S.A.)	2

*Combustion temperature = 300°C, Gas total flow = 30 mL/min. S.A. = Synthetic air*.

Taking as reference the conditions used in previous works (method 1, Table 2; Megías-Sayago et al., 2018) atmosphere composition, time and heating rate were varied to evaluate their influence on average gold particle size. The combustion of PVA in synthetic air is an exothermic process and could favor gold nanoparticles sintering facilitated even more by weak metal-support interaction (Au-C). All steps of the calcination process, separated in heating-combustion-cooling were carried out in low oxygen atmosphere (methods 2, 3, 6) to reduce the exothermicity of the process and minimize gold sintering. Pure nitrogen flow was also used during heating and cooling steps (methods 4 and 5) to improve presumably the annealing process, during which the protective agent is converted in a surface carbon layer covering the gold particle but allowing stronger metal-support interactions and decrease of the sintering rate (Zhan et al., 2017). Oxygen contact time was varied during the calcination step or by decreasing the heating rate (methods 3 and 6).

### Charcoal Powder Support Variation

The optimized synthesis procedure including calcination process described in method 3 was employed to prepare a new series of Au/C catalysts with different charcoal supports. Depending on the hydrophilic properties of the charcoals, different water-ethanol mixtures were used (Table 3). The used charcoals are:

- Carbon obtained by pyrolysis of commercial microcrystalline cellulose in CO_2_ atmosphere (CC) (Santos et al., 2018a).
- Carbon obtained by pyrolysis of wine shoot previously demineralized with nitric acid in CO_2_ atmosphere (CS).
- Carbon obtained after 4 M nitric acid (room temperature, 4 h) treatment of CS sample (CSA).

**Table 3:** Nomenclature and water-ethanol mixtures employed.

**Catalyst**	**Water:ethanol % (v/v)**
Au/CC_8_3	8:92
Au/CC_30_3	30:70
Au/CS_8_3	8:92
Au/CS_30_3	30:70
Au/CS_100_3	100:0
Au/CSA_30_3	30:70
Au/CSA_100_3	100:0

The selected charcoals present different textural and hydrophilic characteristics and allow to investigate the influence of support properties on the final catalyst.

#### Characterization Techniques

XRD analysis were carried out on Panalitycal X'Pert Pro diffractometer, equipped with Cu anode. Diffractograms were recorded with 0.05° step size and 300 s acquisition time in the 10–90° 2θ range.

PHILIPS CM-200 was used for the transmission electron microscopy (TEM) measurements of the initial gold colloids and the final Au/C samples. The average gold particle size is determined taking into account particles surface distribution (Equation 1).

(1) $$\begin{array}{r} D\left[3,2\right]=\frac{\sum_{1}^{n}D_{i}^{3}\nu_{i}}{\sum_{1}^{n}D_{i}^{2}\nu_{i}} \end{array}$$

where Di is the geometric diameter of the *i*th particle, and *vi* the number of particles with this diameter. For every distribution, the number of measured particles overcomes 200 particles per sample.

UV-Vis spectra were acquired on Cary 300 UV-Vis spectrometer equipped with optic fiber liquid sensor for wavelengths ranged between 190 and 900 nm and 5 absorbance units working range to measure turbid and solid liquid samples.

Raman spectra were recorded using dispersive Horiba Jobin Yvon LabRam HR800 microscope with a He–Ne green laser (λ 532.14 nm) working at 20 mW with 600 g mm^−1^ grating, 50× objective and a confocal pinhole of 1,000 μm.

DRIFTS spectra were obtained at room temperature without sample dilution, using a JASCO FTIR 6200 spectrometer equipped with an accessory DRIFTS Pike EASI-DIFF. Each spectrum was recorded accumulating 100 scans with resolution of 4 cm^−1^.

#### Catalytic Tests

For the reduction of 4-nitro to 4-aminophenol at room temperature with NaBH_4_ three separate solutions were prepared: (i) 2.5 mg of catalyst dispersed in 10 mL of water, (ii) 0.39 g of NaBH_4_ dissolved in 10 mL of water, and (iii) 28.4 mg of 4-nitrophenol (Sigma Aldrich, 99%) dissolved in 10 mL of water. A final volume of 30 ml was obtained after contacting all three solutions in the order (i) (catalyst) + (ii) (NaBH_4_) + (iii) (4-nitrophenol), and reaction time 0 was considered. Aliquots samples of 1 mL was extracted at different reaction times and analyzed by UV-Vis spectroscopy, where the evolution of the absorption band centered at 400 nm (corresponding to 4-nitrophenolate ions) was followed until disappearance. AvaLight-DH-S-BAL UV-Vis spectrometer UV-Vis equipped with fiber optic liquid sensor was used for these measurements.

## Results and Discussion

### Influence of the Solvent Nature

The mixture of two solvents with different polarity (water and ethanol in our case) allows the adjustment of the dielectric constant of the medium according to the hydrophilic/hydrophobic behavior of the carbon material. However, the presence of two solvents will influence the colloid behavior and most probably the final gold particle size. In order to evaluate the extension of this effect, UV-Vis study of the colloids obtained in different solvent mixtures was carried out.

**Figure 1:**
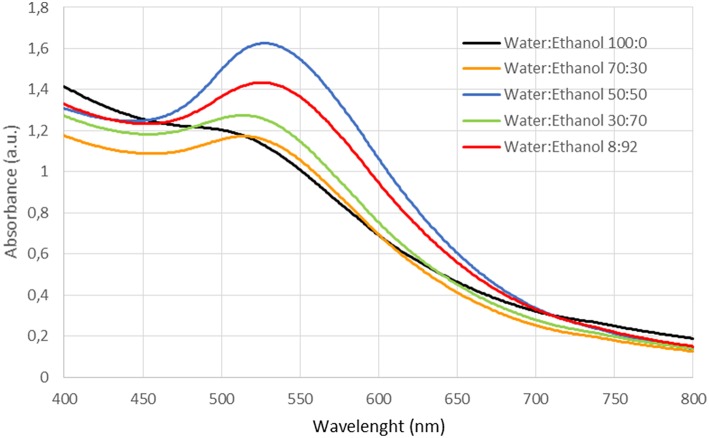
UV-vis spectra of the obtained gold colloids.

The UV-Vis spectra for all colloids show maximum absorption between 520 and 540 nm (Figure 1), corresponding to the characteristic range of surface plasmon resonance (SPR) of gold nanoparticles. It is known that SPR position, area and intensity depend on the size and shape of the gold nanoparticles as well as on the dielectric constant of the medium (Bond and Thompson, 1999).

Generally, an increase in particle size implies a redshift of SPR and an increase in the area of absorption peak (Bond and Thompson, 1999). The estimated values of the wavelength of maximal absorbance and calculated area (400–700 nm range) indicate that a continuous increment of ethanol concentration produces a redshift and SPR area increase, suggesting particle size growth (Table 4). Only the colloid generated at a water:ethanol 50:50 ratio escapes from the tendency.

**Table 4:** Average gold particle size (TEM), maximal absorbance wavelength λ_max_, SPR calculated area, dielectric constant of the used medium.

**Water:ethanol (% v/v)**	**Average gold particle size, TEM (nm)**	**λ_**max**_, SPR (nm)**	**SPR calculated area (a.u.)**	**ε at 25^**°**^C (F/m)**
100:0	2.9 ± 0.7	523	230.84	78.54
70:30	3.6 ± 0.9	531	249.03	62.26
50:50	7.8 ± 1.7	537	339.46	51.41
30:70	4.5 ± 1	531	267.75	40.56
8:92	8.5 ± 2	540	313.27	28.62

The average gold particle size studied by TEM microscopy as a function of solvent nature is resumed in Table 4.

**Figure 2:**
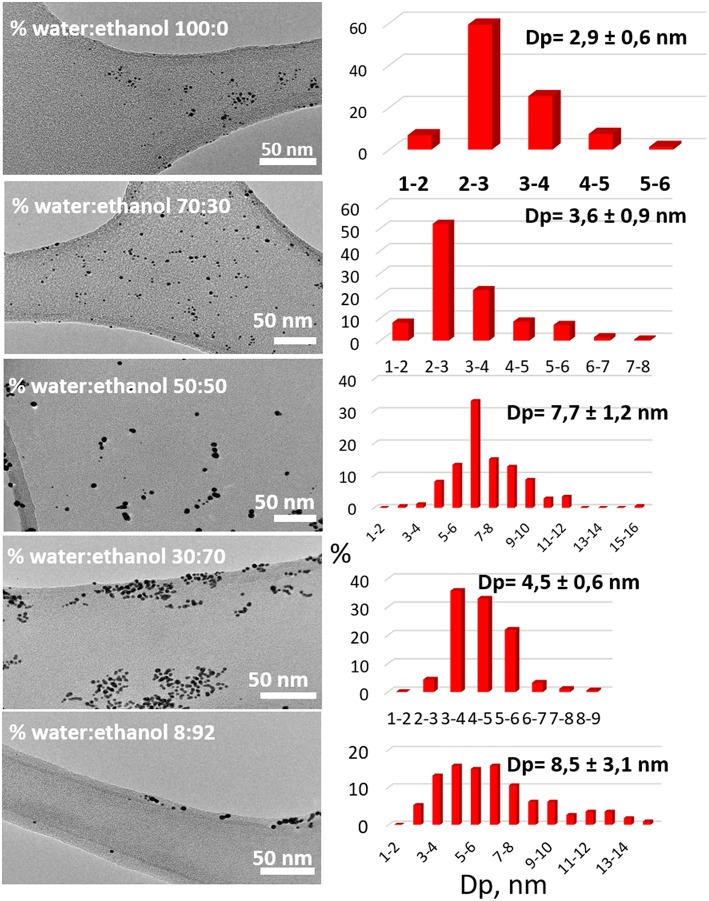
TEM micrographs, particle size distribution, and average gold particle size (Dp) for the obtained colloids.

The tendency observed by UV-Vis is confirmed by TEM, higher the ethanol concentration higher the average particle size (Figure 2) exception made by 50:50 sample, which presents an exceptional higher value. As for the distribution of sizes, the colloids with smaller average particle size present narrower size distribution than those with larger average particle size. The Gaussian distribution becomes less symmetric with the increase of the average particle size. Macroscopically, the colloids with smaller average particle size are reddish, while those with a larger average particle size purple, in agreement with previous studies by Bond and Thompson (1999).

The dielectric constant of the solvents mixture is calculated according to Equation (2) (Jouyban and Soltanpou, 2010) and resumed in Table 4.

(2) $$\begin{array}{r} \varepsilon =\left(\frac{\%water}{100}\cdot \varepsilon_{\text{water}\left(25{}^{\circ}C\right)}\right)+\left(\frac{\%ethanol}{100}\cdot \varepsilon_{\text{ethanol}\left(25{}^{\circ}C\right)}\right) \end{array}$$

**Figure 3:**
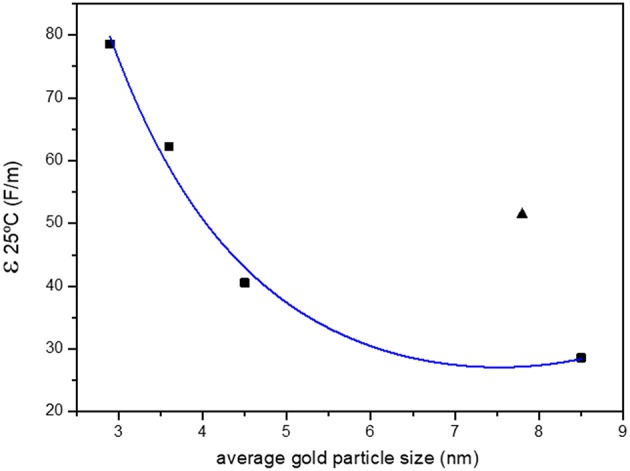
Relationship of the average particle size with the dielectric constant of the solvent.

The dielectric constant increases with the ethanol concentration. The relationship between the constant and gold particle size is presented in Figure 3. It is clear how the dielectric constant of the mixture influences the size of the resulting gold nanoparticles, the lower the percentage of water the higher the average size of the nanoparticles. The colloid 50:50 is out of the described trend, higher particle size is observed contrary to the expected from the calculated dielectric constant. This synthesis was repeated several times, showing similar results. It is necessary to carry out an in-depth study to explain this unexpected result.

**Figure 4:**
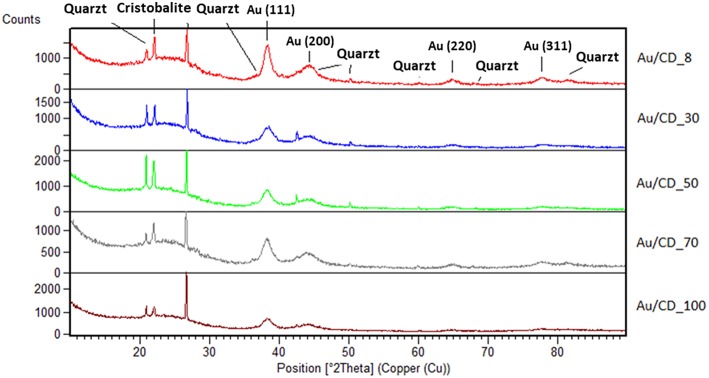
Au/C catalysts diffractograms.

XRD analysis of the Au/C catalysts (Table 1) obtained after gold colloids immobilization are presented in Figure 4. The diffractograms show reflections associated with metallic gold (ICDD 00-001-1172), carbon, quartz (ICDD 01-086-1628), and cristoballite (ICDD 00-004-0379) (the formers present in DARCO^®^ as impurities) (Santos et al., 2018b). The average gold crystallite size was calculated applying Scherrer's equation from the broadening of the (111) Au plane at 38.28° 2θ (Table 5). All samples present average particle size in the 5–7 nm range. According to the average particle size of the colloids, higher the water proportion, lower the gold particle size.

**Table 5:** Gold nanoparticles average size of the Au/C catalysts obtained by TEM and XRD.

**Catalyst**	**Average size, TEM (nm) S&M**	**Average size, XRD (nm)**
Au/CD_100	8.3 ± 1	5.3
Au/CD_70	9.6 ± 1.6	5.9
Au/CD_50	12.1 ± 3.8	6.8
Au/CD_30	7.4 ± 0.7	6.2
Au/CD_8	11.8 ± 1.7	7.0

**Figure 5:**
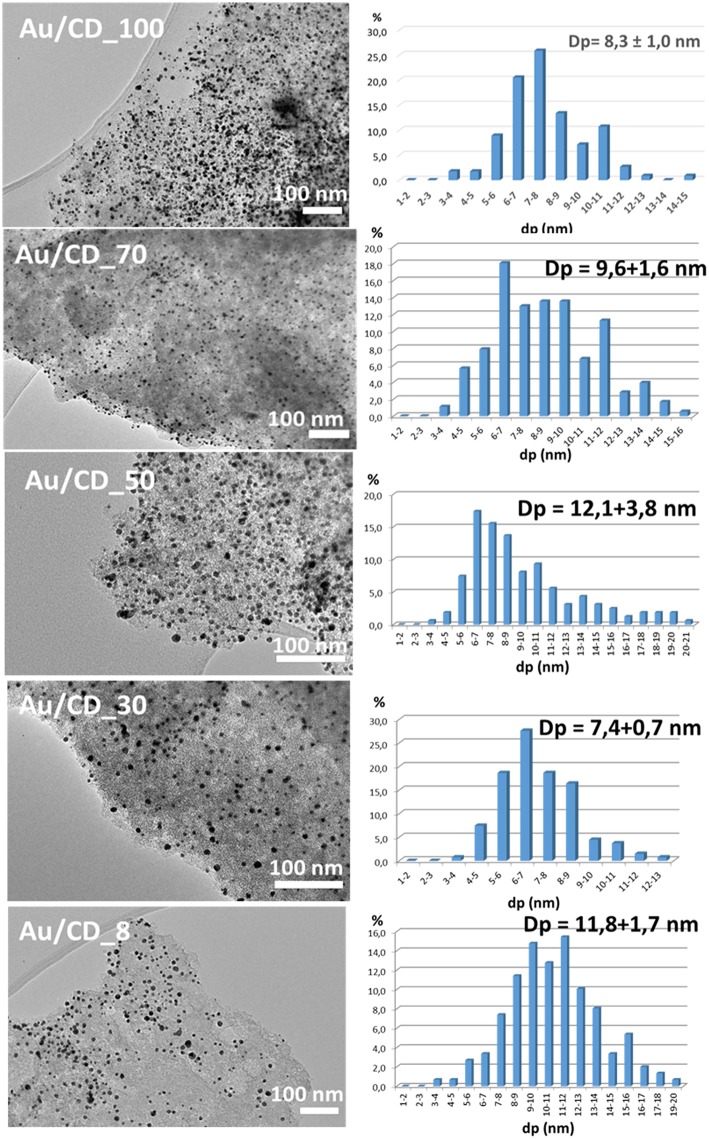
TEM micrographs and gold particle size for Au/C catalysts.

More detailed analysis of the samples by TEM (Figure 5) reveal bigger particle size of the supported nanoparticles than the corresponding colloids, due to the sintering occurring during calcination. It appears that the particle size distributions for the supported samples are less narrow than the one observed for colloids. The tendency observed by XRD and TEM are the same, however, when the size homogeneity is low and the number of measured particles by TEM not high enough, the obtained average size could imply an important error. That is why the Scherrer results are considered for all future discussions.

**Figure 6:**
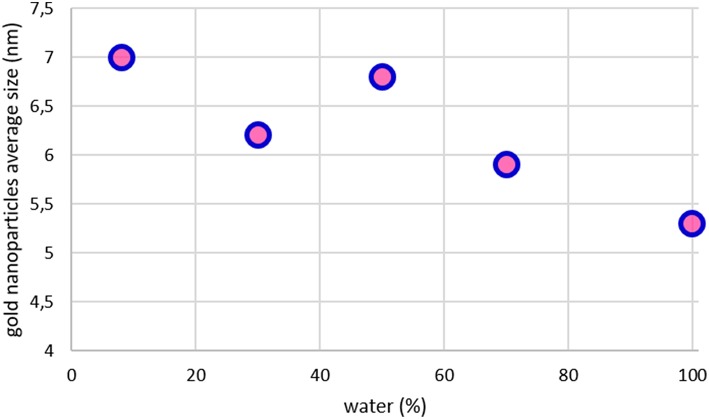
Relationship of the gold average particle size with the water percentage of the solvent.

If we correlate the gold average size (obtained by XRD) with the percentage of water in the solvent mixture (Figure 6), we can see that size decreases when the water content in the mixture increases. This is somehow an expected result, since DARCO^®^ is a hydrophilic carbon that should present stronger interaction with polar solvents, giving smaller particles size at higher water concentration.

The relationship between the gold average particles size in Au/C catalysts (XRD) and in colloids (TEM) is also almost linear, with smaller the colloid size, smaller the size on catalyst surface. The transfer process does not seem to affect seriously the gold nanoparticles size.

### Calcination Process

As explained in section Calcination process, six different calcination processes were applied (Table 2) to optimize the calcination step for the elimination of the protective agent (PVA) in order to obtain the smallest particle size. In all cases, the same Au/C catalyst (Au/CD_100) was calcined at a constant final temperature (300°C) and total gas flow (30 mL/min).

**Figure 7:**
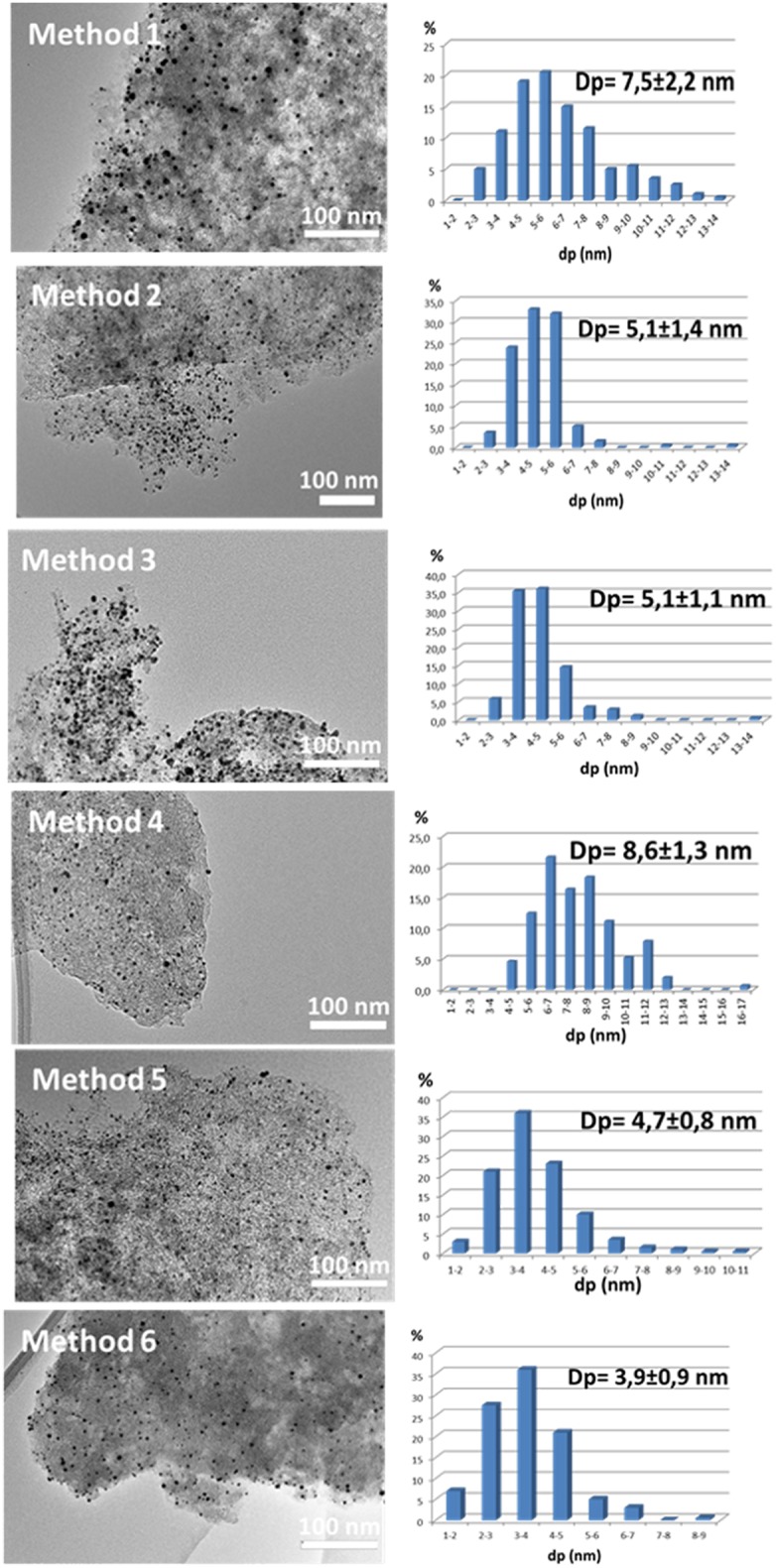
TEM micrographs and gold particle size distribution for Au/C catalysts obtained with different calcination methods.

As showcases in Figure 7 and Table 6, the calcination process clearly influences the gold nanoparticle size. According to the results gold particle size oscillates between 5 and 7 nm and varies with the oxygen concentration and treatment duration. For the same duration of calcination treatment, the particle size decreases proportionally to the oxygen concentration (methods 1 and 2). In fact, samples where the combustion step is carried out in synthetic air (methods 1 and 4) present bigger particle size. Moreover, neither the annealing process (methods 4 and 5) nor the reduction of the heating rate achieves lower particle sizes (methods 3 and 6). Method 3 that implies lower duration, i.e., low energy consumption is selected as the more adequate. In addition, the average particle size obtained for this sample is the smallest, according to TEM and XRD measurements. The application of this calcination procedure reduces sintering phenomena resulting in a minimal increase from 2.9 (colloid) to 4.6 nm (supported catalyst). All samples calcined by the method 3 will be labeled from now on as Name_3.

**Table 6:** Average gold particle size of the Au/C catalyst obtained with different calcination methods.

**Method**	**Average gold particle size, TEM (nm)**	**Average gold particle size, DRX (nm)**
1	7.5 ± 2.2	5.2
2	5.1 ± 1.4	3.9
3	5.1 ± 1.1	3.7
4	8.6 ± 1.3	5.0
5	4.7 ± 0.8	4.2
6	3.9 ± 0.9	4.3

### Au/C Catalyst Over Different Charcoals Powder Support

As described in section Au/C Catalyst over different charcoals powder support, a second series of Au/C catalysts were synthetized employing diverse charcoal powders as supports and compared with Au/CD catalysts.

**Figure 8:**
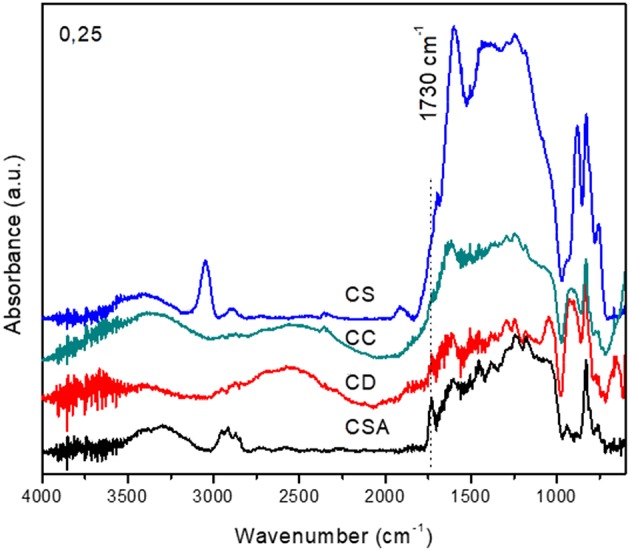
DRIFTS spectra of the employed charcoals.

The supports were selected in order to study the possibility to expand the horizons of the developed synthesis technique for a variety of carbonaceous materials with different hydrophilic character and functionalization degree. DRIFTS spectroscopy (Figure 8) was used for such evaluation, since it is able to detect the oxygenated functional groups on the carbon surface, providing determinant information about the surface chemical structure of the material (Meldrum and Rochester, 1991; Fanning and Vannice, 1993; Wang et al., 2019).

Along with the IR bands of aromatic and aliphatic carbons (C = C stretching vibration, 1,600 cm^−1^, C-C stretching vibrations, 1,450 cm^−1^, C-H of aliphatic chains around 2,900 cm^−1^), C-H of aromatic compounds (3,040 cm^−1^), and C-H out of plane vibrations of substituted benzene rings (750 and 950 cm^−1^) (Lin-Vien et al., 1991), all solids present bands characteristics of several oxygenated functional groups. Those bands, in the 1,030–1,300 cm^−1^ region, are indicative of C-O single bond of different oxygenated groups such as esters (1,150–1,250 cm^−1^), acidic and cyclic anhydrides (1,180–1,300 cm^−1^), lactones (1,160–1,370 cm^−1^), ethers (1,120–1,300 cm^−1^), cyclic ethers (1,140 cm^−1^), phenolic groups (1,180–1,220 cm^−1^), epoxides (1,220 cm^−1^), etc. Besides this, an intense band a 1,730 cm^−1^ due to C = O aldehyde groups is clearly visible. In the case of the CC charcoal, a band centered at 1,900 cm^−1^ corresponding probably to lactones (Lin-Vien et al., 1991; Meldrum and Rochester, 1991; Fanning and Vannice, 1993; Wang et al., 2019) is also detected, together with very intense bands of substituted aromatic rings (3,040 cm^−1^, between 1,000 and 1,300 cm^−1^ and between 750 and 950 cm^−1^).

The wide band between 3,200 and 3,600 cm^−1^ is due probably to the O-H vibrations of water adsorbed on the superficial functional groups. In order to classify the compounds according to their degree of functionalization (Daud and Houshamnd, 2010), the band at 1,730 cm^−1^ of the carbonyls groups was taken as reference. The intensity of this band could be correlated to the degree of superficial hydrophilicity, i.e., high intensity of the band corresponds to greater functionalization and hydrophilia. The hydrophilicity decreases in order: CSA > CD > CS > CC.

This is a reasonable order, taking into account the different provenance and nature of the studied charcoals. Thus, the CC one obtained by pyrolysis in CO_2_ atmosphere from commercial microcrystalline cellulose (Santos et al., 2018a), without any kind of functionalization, or presence of inorganic material, present a marked hydrophobic character. The CS sample, obtained by a similar pyrolysis process, comes from a natural lignocellulosic biomass (vine shoot), with different oxygen content and inorganic compounds in its structure. Prior to the pyrolysis CS sample undergoes a demineralization treatment with nitric acid, which contributes to a certain oxidation of the organic matter. It can be expected, therefore, that the obtained carbon presents higher oxygen content, reflecting in higher hydrophilicity. Finally, both, commercial activated carbon (CD) and CSA charcoals, activated after pyrolysis in nitric acid, contain greater percentage of oxidized surface groups, and consequently greater hydrophilic character.

Based on the observed hydrophilic properties of the samples, different water-ethanol mixtures were used (Table 3) to prepare the second series of catalysts, high ethanol concentration for the most hydrophobic ones and *vice versa*.

The average gold particle size of the catalysts measured by XRD is shown in Table 7. Very interesting observation arises, the hydrophobicity of the support is directly related to the solvent mixture and to the final particle size. As long as more ethanol is used for the hydrophobic samples (Au/CC_8_3 vs. Au/CC_30_3) and more water for the hydrophilic ones (Au/CSA_30_3 vs. Au/CSA_100_3) the average particle size decreases. Even more, for Au/CSA_100_3 sample no gold diffractions are observed indicating gold average particle size below the detection limit of the technique. For CS, which has an intermediate hydrophilic character, no obvious correlation is observed.

**Table 7:** Average gold nanoparticles size for the Au/C catalysts.

**Catalyst**	**Gold nanoparticles size XRD, nm**
Au/CC_8_3	7.2
Au/CC_30_3	8.0
Au/CS_8_3	5.5
Au/CS_30_3	6.2
Au/CS_100_3	4.6
Au/CD_100_3	3.7
Au/CSA_30_3	8.7
Au/CSA_100_3	–

Comparing the average gold particles size for the catalysts synthetized in water only for different charcoals as supports, the sizes sequence follows the hydrophilicity degree: Au/CSA_100_3 (below the detection limit) < Au/CD_100_3 (3.7 nm) < Au/CS_100_3 (4.6 nm).

The results evidence that, despite that the alcohol media disfavors obtaining of gold colloids with lower sizes, its use can be beneficial if the carbonaceous material presents an important hydrophobic character.

#### Catalytic Tests: 4-Nitrophenol Reduction

For the reduction of 4-nitrophenol, two samples from the first series are used Au/CD_100_3 and Au/CD_8_3 as representatives of the most different gold average particle size, 6.5 and 11.8 nm, respectively. The selected catalysts are useful to study how the gold particle size influences the rate constant of the reaction, with the 4-NP to 4-AP transformation monitored by UV-Visible absorption spectroscopy.

**Figure 9:**
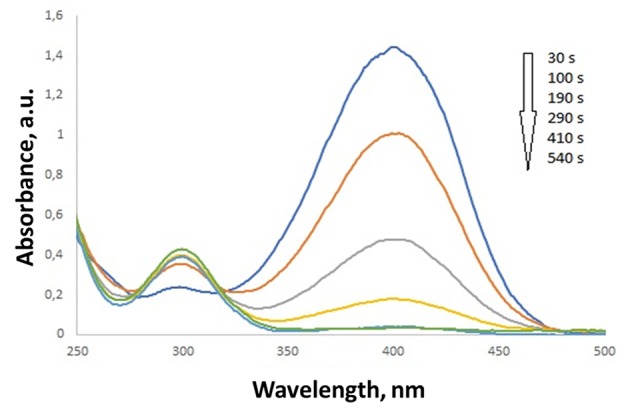
Evolution of the UV-vis absorption spectra during the reduction of 4-NP.

The characteristic absorption peak of 4-NP is located around 317 nm. When NaBH_4_ is added to 4-NP, in absence of catalyst, a color modification of the solution from light to dark yellow is immediately observed, accompanied by a shift in the peak position from 317 to 400 nm. This evolution is associated with the formation of 4-nitrophenolate anions (Berahim et al., 2018). When catalyst is present into the solution the absorption at 400 nm decreases, while a new signal around 300 nm appears, increasing with time and associated with the reaction product, 4-AP (Figure 9). The presence of the isosbestic point around 320 nm, indicates the reduction of 4-NP to 4-AP occurs without by-product formation (Berahim et al., 2018).

Since the concentration of ${\text{BH}}_{4}^{-}$ is much higher than that of 4-NP, it can be assumed that the borohydride concentration remains constant during the reaction (Berahim et al., 2018) and the reaction follows pseudo-first order kinetics given by the Equation (3),

(3) $$\begin{array}{r} \frac{C}{C_{0}}=\exp\left(-kt\right) \end{array}$$

where C_0_ and C represent the initial and final concentrations of 4-NP, t symbolizes the reaction time and k denotes the rate constant.

**Figure 10:**
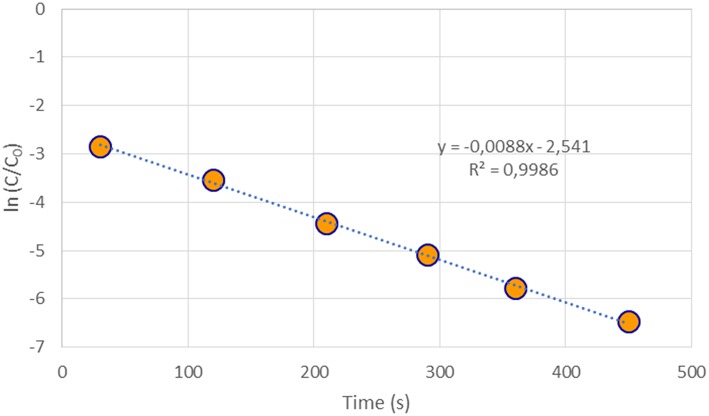
ln (C/C_0_) vs. time for 4-NP reduction using Au/CD_8 as catalyst.

To calculate the rate constant of the 4-NP reduction reaction, the variation of its concentration with time is studied. A linear correlation is observed between ln(C/C_0_) and time, obtaining the rate constant from the slope of this graph (Figure 10). Three catalytic tests are realized for each sample, showing high reproducibility. The calculated rate constant is obtained after averaging the three values.

For the Au/CD_8_3 catalysts, with an average gold particle size of 11.8 nm (7 nm XRD), the value of the obtained rate constant is *k* = 8.90 × 10^−3^ s^−1^ (0.534 min^−1^), while for the Au/CD_100_3, with size average of 8.3 nm (5.3 nm XRD), rate constant increases to *k* = 1.10 × 10^−2^ s^−1^ (0.660 min^−1^). Hence the impact of gold particle size in the reaction kinetics is clearly evidenced in view of these results. Essentially smaller the gold nanocrystals size higher the increase the 4-NP reduction rate. The rate constant obtained for both catalysts present superior values for similar noble based catalyst (Table 8). It appears that the carbonaceous support is not as important as gold particles size or doping. The presence of graphene oxide does not promote the gold action and for a similar size the use of activated carbon seems better (our study), probably due to the higher surface area of the latter. On the other hand, the doping of gold with silver presents a beneficial effect and results in higher rate constants.

**Table 8:** Comparison of the catalytic reduction of 4-NP by different noble metal catalysts.

**Catalyst**	**Rate constant (min^**−1**^)**	**Particle size (nm)**	**References**
Au/CD_8_3	0.534	7.0	Reference
Au/CD_100_3	0.660	5.3	This work
Au NPs	0.360	24.1	This work
Au-Ag NPs	0.620	55.2	Berahim et al., 2018
Ag/GO NPs	0.208	7.5	Berahim et al., 2018
Au/GO NPs	0.368	5.0	Wu et al., 2013
Au-Ag/GO NPs	0.761	6.0	Wu et al., 2013
Pd-graphene nanohybrids	0.197	17.0	Wang et al., 2014

The reaction mechanism (Langmuir-Hinshelwood) is frequently related to the electron transfer from NaBH_4_ to 4-NP adsorbed on the same active site (Wunder et al., 2010, 2011). The metal surface assures the necessary sites for adsorption and transfer of electrons. It is then logical to consider that greater the exposed surface area higher the rate of electron transfer and higher the rate constant of 4-NP reduction.

It is clear, in our case that the reaction rate depends on the number of exposed gold sites, for gold particles on the same support. On the other hand, the gold dispersion depends on the optimization of the synthetic parameters as solvent, support nature and calcination procedure. Although the gold colloid preparation appears as an easy task, its immobilization is much more complicated. The nature of the support when referring to carbon is one of the most important parameters to take into account. Hydrophobic carbons force the use of less polar solvents as ethanol, which reflects in an increase of the initial colloid size as the latter is smaller when more polar solvents are used (water). Referring to the calcination procedure the most important parameter is the atmosphere of calcination more precisely the oxygen concentration. Lower the oxygen concentration, lower the particle size. The heating rate and duration are not so relevant and could be chosen as compromise between size and energy and time savings.

## Conclusions

This work showcases a flexible strategy to prepare nanogold based catalysts via colloids transfer to suitable supports—activated carbons in particular. Water: Ethanol mixtures has been selected as reference solvents for the synthesis. Indeed, there is a remarkable impact of solvent on the average gold particle size in the colloids in such a way that when the amount of ethanol in the solvent mixture increases, the average gold particle size increases. An anomalous behavior is observed for a 50:50 mixture. In fact, the affinity of the support for water is also a factor to consider where the solvent can tune the resulting catalysts' features. Depending on hydrophilicity of the support the water:ethanol ratio could be adjusted to obtain the smallest average gold particle size.

A successful transfer of the colloid nanoparticles to commercial hydrophilic activated charcoal is obtained. The final gold particle size is superior to that of colloids due to sintering, with the smallest one obtained for the Au/CD_100 (prepared in water).

Oxygen presence in the thermal treatment also has a marked effect on the resulting catalysts. We observed that tower the concentration of oxygen during the treatment lower the particle size and hence higher exposed active surface for the reaction. Among the different thermal treatments carried out in order to remove the organic protective agent, PVA, the optimal one is: 300°C, an oxidative flow of 30 mL/min of N_2_-synthetic air mixture (2% S.A.) for 30 min and a heating rate of 10°C/min.

Finally, the as prepared gold based catalysts has demonstrated their efficiency for environmentally relevant catalytic reactions i.e., the reduction of 4-NP. In the process, the average gold particle size of the Au/C catalyst influences the kinetics of the reaction, in such a way that, smaller the gold particle size, higher the constant rate.

## Data Availability

The datasets generated for this study are available on request to the corresponding author.

## Author Contributions

All authors listed have made a substantial, direct and intellectual contribution to the work, and approved it for publication.

### Conflict of Interest Statement

The authors declare that the research was conducted in the absence of any commercial or financial relationships that could be construed as a potential conflict of interest.
